# Microbial Composition of Carious Dentin and the Impact of Minimally Invasive Excavation Techniques: A Narrative Review

**DOI:** 10.3390/ijms27135648

**Published:** 2026-06-23

**Authors:** Nadezhda Mitova, Zornitsa Lazarova

**Affiliations:** Department of Pediatric Dentistry, Faculty of Dental Medicine, Medical University—Sofia, 1 Georgi Sofiyski St., 1431 Sofia, Bulgaria; z.lazarova@fdm.mu-sofia.bg

**Keywords:** dental caries, dentin, microbiota, biofilms, minimally invasive dentistry, microbiological techniques, molecular diagnostic techniques, photodynamic therapy, caries removal

## Abstract

Dental caries is a biofilm-mediated dysbiotic disease characterized by ecological shifts within the oral microbiome and progressive demineralization of dental hard tissues. The microbiological composition of carious dentin and the impact of minimally invasive excavation techniques on residual microbial communities remain subjects of ongoing investigation due to methodological heterogeneity and inconsistencies among published studies. This narrative review aimed to summarize current evidence regarding the microbial ecology of carious dentin, compare culture-based and molecular methods for microbiological assessment, and evaluate the microbiological outcomes associated with contemporary approaches to managing minimally invasive caries. The relevant literature on dentinal caries microbiology, microbial detection methods, and excavation techniques was analyzed. The available evidence indicates that carious dentin contains a highly diverse polymicrobial community composed of acidogenic, aciduric, anaerobic, and proteolytic microorganisms. Culture-based methods primarily detect viable and cultivable taxa, whereas molecular approaches reveal substantially greater microbial diversity, including uncultivable and low-abundance species. Comparative studies demonstrate that minimally invasive excavation techniques significantly reduce microbial load but rarely achieve complete microbial elimination. The available evidence suggests that successful caries management is associated with a reduction in and ecological modulation of the residual microbiota within a sealed environment. The integration of culture-based and molecular findings provides a more comprehensive understanding of the microbiology of carious dentin and supports biologically oriented, minimally invasive strategies for caries management.

## 1. Introduction

In the contemporary literature, dental caries is defined as a biofilm-induced, dysbiotic disease characterized by ecological changes in microbial communities and subsequent demineralization of the hard dental tissues [1,2]. Within this concept, known as the ecological plaque hypothesis, dental caries is regarded as the result of a functional reorganization of the oral microbiome, in which stable, acid-tolerant microbial consortia with increased cariogenic potential are formed [3,4].

Carious dentin is a microbially and structurally altered hard tissue that develops as the lesion progresses into deeper tooth structures, in which a complex ecosystem is formed, consisting of bacteria, a degraded collagen matrix, and dentinal tubules. Contemporary studies have shown that even in deep lesions, the microbial composition can be highly variable, exhibiting active bacterial populations and areas with limited or absent bacterial colonization [5]. This heterogeneity reflects the dynamic nature of the lesion, with important clinical implications for caries removal strategies, particularly regarding the extent of infected tissue that may still harbor viable microorganisms [6,7].

From a clinical and histopathological perspective, the distinction between infected and affected dentin is significant. Infected dentin is characterized by a high bacterial load, pronounced structural disorganization, a soft consistency, and irreversible degenerative changes in the collagen matrix, which necessitate its removal [6,8]. In contrast, affected dentin shows significantly lower bacterial colonization, with contemporary studies often reporting the presence of single, low-abundance, or undetectable microorganisms, together with preserved structural integrity, a firmer consistency, and the potential for remineralization [9,10].

Despite significant advances in molecular microbiology, including polymerase chain reaction (PCR)-based and sequencing approaches, the results of different studies remain heterogeneous. Differences between culture-dependent and independent methods lead to inconsistencies in reported microbial diversity, particularly in deep dentinal lesions, where sampling accessibility and oxygen conditions vary considerably. Culture-based and molecular approaches often demonstrate different sensitivities and coverage, which complicates direct comparisons between studies and interpretation of microbial profiles [8,11].

In addition, contemporary systematic reviews have shown that findings regarding residual bacterial load remain contradictory, even when standardized clinical methods for selective carious dentin removal are applied [12,13]. Studies have shown that residual bacterial presence may persist even after partial or complete caries removal [14]. This highlights the lack of consensus regarding microbial persistence following different therapeutic interventions.

Recent advances in minimally invasive caries management include chemo-mechanical caries removal systems, laser-assisted techniques, and photodynamic therapy. These approaches aim to selectively remove infected dentin while preserving sound tooth structure and reducing microbial load, and they are increasingly supported by contemporary evidence on caries management [15].

The absence of a unified microbial profile of carious dentin, the contradictory evidence regarding the effect of minimally invasive techniques on microbial reduction, and existing methodological and conceptual inconsistencies indicate a significant gap in the current literature. This underscores the need for an integrative approach combining clinical, culture-based, and molecular data to achieve a more precise understanding of the microbial ecology of the carious process [16,17].

This narrative review provides a comprehensive overview of the microbiological aspects of carious dentin. First, the microbial ecology of carious lesions is discussed, emphasizing the composition and functional organization of the caries-associated biofilm, followed by an analysis of culture-based and molecular diagnostic approaches used to characterize the oral microbiota. Subsequently, the microbiological impact of minimally invasive caries excavation techniques is presented, including mechanical, chemo-mechanical, laser-assisted, and photodynamic strategies. Finally, this review integrates the current findings to highlight methodological limitations, clinical implications, and future perspectives in the field of caries microbiology.

### Literature Search Strategy

This narrative review was based on a targeted literature search conducted in PubMed, Scopus, and Google Scholar to identify relevant publications addressing the microbiology of carious dentin and the microbiological effects of minimally invasive caries excavation techniques. The search focused primarily on articles published between 2000 and March 2026, with particular emphasis on recent studies addressing contemporary concepts of oral microbiome ecology and minimally invasive caries management.

The search included combinations of the following keywords: “carious dentin”, “dental caries microbiology”, “oral microbiome”, “caries-associated biofilm”, “culture-based methods”, “PCR”, “16S rRNA sequencing”, “minimally invasive dentistry”, “selective caries removal”, “chemo-mechanical caries removal”, “BRIX 3000”, “Carisolv”, “Papacárie”, and “photodynamic therapy”.

Original research articles, clinical studies, systematic reviews, meta-analyses, and relevant narrative reviews published in English were considered. Publications were selected based on their relevance to this review’s objectives, with particular focus on studies investigating the microbial composition of carious dentin, microbiological assessment methods, and the effects of different excavation techniques on residual microbiota.

Studies were excluded if they were not relevant to the microbiological aspects of carious dentin or minimally invasive caries management; not published in English; or lacked sufficient methodological detail regarding microbial assessment. Duplicate publications and studies with insufficient data on microbial outcomes were also excluded from the analysis.

## 2. Microbial Ecology of Carious Dentin

The microbial composition of carious dentin is characterized by high taxonomic diversity and a dynamic polymicrobial organization, reflecting the complex ecology of the carious process. Contemporary concepts of oral dysbiosis suggest that the development of dentinal carious lesions is caused by functional interactions between acidogenic, aciduric, proteolytic, and anaerobic microbial communities within the caries-associated biofilm, rather than by a single pathogenic microorganism [3,18,19].

In the initial developmental stages of dentinal carious lesions, Gram-positive acidogenic microorganisms predominate, primarily members of the genera *Streptococcus* and *Lactobacillus*. *Streptococcus mutans* remains one of the most extensively studied cariogenic microorganisms due to its ability to produce organic acids, form an extracellular matrix, and adapt to low pH conditions [19]. This microbial shift reflects the selection of a core cariogenic microbiota associated with lesion progression under sustained acidic conditions [20].

However, molecular identification methods have demonstrated that the microbial composition of dentinal lesions is significantly more diverse compared with results obtained using classical culture-based techniques [20,21]. An increased relative abundance of anaerobic and proteolytic taxa is observed when lesions progress into deeper dentin layers, including members of the genera *Prevotella*, *Veillonella*, *Fusobacterium*, *Actinomyces*, *Scardovia*, and *Bifidobacterium* [22,23]. Sequencing studies of deep carious lesions have shown that these microorganisms are associated with the degradation of the organic matrix and the maintenance of an acidic microenvironment, thereby promoting disease progression [24]. Selvakumar et al. demonstrated the presence of active bacterial populations in carious dentin under different pulpal conditions [5].

Significant differences have been reported between the microbial profiles of infected and affected dentin. Infected dentin is characterized by a high bacterial density, extensive invasion of dentinal tubules, and pronounced degradation of the collagen matrix [6,25]. In contrast, affected dentin is generally characterized by limited bacterial populations. Several contemporary studies have reported low-abundance microorganisms or levels below the detection threshold, depending on the methodology used, following the removal of selective carious tissue [12,25,26].

In addition to lesion depth and tissue characteristics, the topographical location of carious lesions may also influence their microbial ecology. Cervical and root surface lesions have been associated with distinct ecological conditions and a greater prevalence of *Actinomyces* spp. and other microorganisms adapted to exposed root surfaces. These site-specific differences further contribute to the heterogeneity of microbial profiles reported in carious dentin and should be considered when interpreting microbiological findings [16,23].

The methodology used for microbiological identification substantially influences the microbial profile detected. Classical culture-based techniques allow the assessment of viable microorganisms and antimicrobial susceptibility; however, they remain limited regarding fastidious and strictly anaerobic taxa. In contrast, PCR-based and 16S ribosomal ribonucleic acid (16S rRNA) sequencing approaches considerably expand the spectrum of detectable microorganisms and enable the identification of microbial populations that remain undetectable using conventional culture-based methods [1,3,27].

Despite significant advances in oral microbiome research, a unified microbial profile of carious dentin has not yet been established. Variations in lesion depth, sampling protocols, molecular detection methods, and clinical excavation techniques contribute to considerable heterogeneity reported across studies [4,24]. Therefore, the integration of culture-based and molecular data remains essential for achieving a more comprehensive understanding of the microbial ecology of dentinal carious lesions [3].

The microbial ecology of carious dentin is characterized by a complex and dynamic consortium of bacterial and, in some cases, fungal species that contribute differentially to lesion initiation, progression, and maintenance. Rather than acting as isolated pathogens, these microorganisms function within structured biofilm communities, where metabolic interactions, acid production, and proteolytic activity collectively drive tissue degradation. The relative abundance and functional roles of these taxa vary depending on lesion depth and ecological conditions, reflecting the progressive nature of the carious process. A summary of the principal microorganisms associated with carious dentin and their proposed roles is presented in Table 1. This ecological complexity highlights the need for a structured overview of the dominant taxa and their context-dependent roles in carious dentin.

The microbial taxa summarized in Table 1 represent key members of the caries-associated biofilm and reflect the ecological succession occurring during lesion progression. Early stages are dominated by acidogenic and aciduric species, whereas deeper lesions are characterized by an increased prevalence of anaerobic and proteolytic taxa adapted to nutrient-limited and acidic environments [16,17,20,24]. These microorganisms do not act as isolated pathogens but rather function within a metabolically cooperative biofilm, where synergistic interactions between microbial communities contribute to sustained acidification and progressive degradation of the dentinal matrix [2,3,18,23]. The functional relevance of these taxa is further supported by histopathological and microbiome-based studies demonstrating coordinated biofilm activity in carious dentin [6,20,24]. However, the reported distribution of microbial species is also influenced by methodological factors, including sampling strategy and identification techniques, which may differentially affect the detection of fastidious, low-abundance, or non-cultivable taxa [11,12,14,27].

In addition to microbial composition and spatial heterogeneity, the ecological behavior of the caries-associated biofilm is strongly influenced by environmental and dietary factors. Dietary fermentable carbohydrates, particularly sucrose and other free sugars, play a central role in modulating the ecological behavior of caries-associated biofilms. Frequent sugar exposure provides a readily metabolizable substrate that favors acidogenic and aciduric species, thereby intensifying organic acid production and maintaining prolonged periods of low pH within the biofilm matrix. This sustained acidification contributes to ecological dysbiosis by selecting for acid-tolerant microorganisms and suppressing health-associated taxa. Even in the presence of residual microbiota within infected or affected dentin, continued exposure to fermentable carbohydrates can sustain metabolic activity and promote a microenvironment conducive to ongoing demineralization. Therefore, substrate availability represents a key ecological driver of biofilm stability and caries progression within the dentinal lesion [16,23].

However, understanding microbial composition alone is insufficient without considering how these microorganisms are detected in different methodological frameworks.

## 3. Culture-Based Characterization of the Carious Dentin Microbiota

Culture-based methods represent one of the earliest and most established approaches for the microbiological investigation of carious dentin. Despite the significant advancement of culture-independent molecular techniques, these methods remain relevant due to their ability to detect viable and metabolically active microorganisms, enable quantitative assessment of bacterial load, and allow evaluation of antimicrobial susceptibility profiles. In the context of carious dentin, cultivation approaches provide functional information regarding the viability and potential pathogenicity of recovered taxa, which cannot be directly inferred from molecular detection alone [13,27,28].

Classical culture-based studies of carious dentin lesions have traditionally demonstrated a predominance of acidogenic and aciduric microorganisms, particularly members of the genera *Streptococcus* and *Lactobacillus*. An increased representation of anaerobic and proteolytic taxa has been observed with lesion progression into deeper dentin layers, reflecting shifts in local oxygen availability and progressive degradation of the organic dentinal matrix [1,29]. These pooled analyses further indicate that *Streptococcus mutans* remains significantly associated with caries-active sites, while *Lactobacillus* spp. show increased abundance in deep, low-pH dentinal environments, supporting a model of ecological succession rather than single-pathogen etiology [12,30]. Overall, contemporary meta-analytic evidence reinforces that dentinal caries progression reflects a structured microbial transition from early acidogenic dominance to anaerobic, proteolytic biofilm communities in advanced lesions [4,12].

Despite these limitations, culture-based methods remain valuable for assessing the clinical effectiveness of different carious dentin excavation strategies. Several recent clinical and systematic investigations have demonstrated that minimally invasive techniques, including selective caries removal and chemo-mechanical excavation, result in variable but significant reductions in viable microbial load, which can be objectively quantified using cultivation-based analyses [12,18,25,26]. These methods provide functional information on residual bacterial viability, which remains clinically relevant for evaluating treatment outcomes and the ecological stability of sealed lesions. Recent systematic reviews of minimally invasive caries management confirm that substantial microbial reduction combined with lesion sealing is associated with favorable clinical outcomes and arrest of lesion progression [25,26]. This supports the continued relevance of culture-based assessments as a complementary tool alongside molecular diagnostics in caries research.

Several classical and contemporary studies have demonstrated that residual viable microorganisms can be detected even after clinically completed carious dentin excavation. Lager et al. reported the presence of cultivable bacteria following conventional bur excavation and chemo-mechanical caries removal using Carisolv [11]. Similar findings have been observed in studies evaluating atraumatic restorative treatment (ART), where residual bacteria were detected within dentinal tubules despite the clinically acceptable hardness of the cavity floor [31]. More recent randomized clinical trials investigating partial caries removal strategies have also shown substantial reductions in bacterial load after selective excavation, without significant differences between minimally invasive approaches regarding residual microbial presence [14].

Recent investigations evaluating minimally invasive techniques in primary teeth have demonstrated significant reductions in cariogenic microbiota following chemo-mechanical caries removal and adjunctive antimicrobial approaches. Recent clinical studies evaluating BRIX 3000 and BRIX 3000 combined with photodynamic therapy reported significant reductions in bacterial load in primary teeth [32,33]. In addition, comparative analyses of culture-based and PCR-based methods in our investigations indicate that cultivation techniques tend to identify a narrower spectrum of microorganisms compared with molecular approaches, particularly in low bacterial loads and mixed anaerobic populations. Related work on permanent teeth has reported similar methodological discrepancies between culture and molecular detection approaches, further supporting the presence of technique-dependent bias in microbiological profiling of carious dentin [34]. These findings underscore that culture-based methods, despite their inherent limitations, remain an essential component of comprehensive microbiological assessment of carious dentin, especially when integrated with molecular diagnostic techniques.

The application of culture-based methods across different caries excavation strategies has been widely investigated in clinical and experimental settings. Key studies evaluating microbiological outcomes following different excavation approaches in carious dentin are summarized in Table 2.

Although the included studies consistently demonstrate a reduction in cultivable bacterial load using different excavation strategies, their substantial methodological heterogeneity limits their direct comparison. Differences in lesion depth, excavation endpoints, and culture conditions significantly influence reported outcomes. More invasive excavation does not consistently correlate with greater microbial reduction, suggesting that the effectiveness of caries management is primarily determined by ecological disruption and sealing quality rather than the extent of hard tissue removal alone. Consequently, microbial reduction should be interpreted as a relative and context-dependent outcome rather than an absolute measure of treatment success [7,12,26,36].

These limitations have driven the adoption of culture-independent molecular approaches for more comprehensive characterization of the caries-associated microbiome. Building upon the limitations of culture-based methods, molecular approaches have further expanded the understanding of the carious microbiome.

## 4. Molecular Approaches for Microbiome Analysis in Carious Dentin

Molecular methods for oral microbiome analysis have substantially reshaped the understanding of microbial ecology in carious dentin by overcoming the intrinsic limitations of culture-based techniques and enabling the detection of uncultivable and fastidious microbial taxa. Contemporary studies of the oral microbiome indicate that culture-dependent approaches capture only a fraction of the true microbial diversity due to the strict growth requirements of many oral bacteria, particularly their dependence on anaerobic conditions and specific nutritional environments [29,37].

Among molecular approaches, PCR, including quantitative PCR (qPCR) and targeted 16S rRNA gene analysis, remains one of the most widely applied techniques for detecting caries-associated microorganisms in dentinal lesions. PCR-based methods demonstrate significantly higher sensitivity compared with culture-based techniques, particularly in deep carious dentin, where acidic, anaerobic, and nutrient-limited conditions select for metabolically adapted microbial communities [1,38].

PCR detects deoxyribonucleic acid (DNA) signatures of a broad range of microorganisms, including key anaerobic and proteolytic taxa such as *Prevotella*, *Veillonella*, *Fusobacterium*, *Scardovia*, and *Bifidobacterium*, which are frequently underrepresented or undetectable using conventional cultivation approaches. Consequently, culture-based methods systematically underestimate the complexity and diversity of the caries-associated microbiota, capturing only a restricted subset of the oral microbial community [24,39,40,41].

A major limitation of PCR-based methods is the inability to distinguish between viable and non-viable bacterial cells. This is particularly relevant in dentin samples collected after caries excavation, where bacterial DNA may persist following the elimination of viable microorganisms, potentially leading to an overestimation of the clinically active microbial population [5]. In this context, methodological modifications, such as viability PCR–propidium monoazide quantitative PCR (PMA-qPCR), have been proposed. However, these approaches remain methodologically sensitive and are not yet fully standardized [42,43].

The development of 16S rRNA amplicon sequencing has significantly expanded the possibilities for microbiological characterization of carious dentin. This approach enables the simultaneous identification of hundreds of bacterial taxa within a single sample and reveals highly heterogeneous yet functionally organized microbial consortia. Sequencing studies have shown that deep carious lesions contain diverse anaerobic and acidogenic communities, with compositions that vary substantially between individuals [40,44].

Systematic reviews of the oral microbiome indicate that dental caries is not associated with a single specific pathogen but rather results from ecological imbalance (dysbiosis) within the microbial community [28,36,44]. This supports the “ecological plaque hypothesis,” according to which the disease arises from a functional reorganization of the microbial consortium rather than the presence of a single dominant pathogen [5,11].

The most advanced approach, shotgun metagenomics, enables simultaneous taxonomic and functional profiling of microbial communities. In contrast with 16S rRNA amplicon sequencing, this method provides direct information on metabolic pathways, including acid production, biofilm formation, proteolytic activity, and carbohydrate metabolism, all of which are critical for the progression of carious lesions [41,45,46,47].

Comparative studies across culture-based, PCR-based, and sequencing approaches demonstrate substantial discrepancies in the reported microbial profiles. Culture-based methods predominantly identify Streptococcus and Lactobacillus species, whereas molecular techniques reveal a considerably broader diversity of anaerobic and proteolytic taxa. This has led to the concept of a “method-dependent microbiome,” in which the observed microbial composition is influenced by the underlying biological community and the analytical methodology employed [44,48,49].

These differences are significant from a clinical perspective, as they affect the interpretation of residual microbial load after caries excavation and the assessment of the efficacy of minimally invasive treatment. None of the individual culture-based, PCR-based, or sequencing approaches provides a complete and self-sufficient representation of the microbial ecology of carious dentin. Therefore, an integrative approach combining culture-dependent, molecular, and functional data is recommended to achieve a more accurate biological and clinical interpretation of the carious process [20,41,47].

Culture-based, PCR-based, and sequencing approaches collectively provide complementary but incomplete perspectives on the microbiological composition of carious dentin. Culture-based methods primarily reflect viable and cultivable taxa, whereas molecular techniques expand detection to include uncultivable and low-abundance microorganisms. However, each methodological approach is associated with inherent biases that influence the observed microbial profile. Therefore, an integrative methodological framework combining culture-dependent, molecular, and functional approaches is required to achieve a more accurate characterization of the caries-associated microbiome [29,37,47,48].

Table 3 summarizes the comparative characteristics of culture-based and molecular methods.

Culture-based methods, PCR-based approaches, and sequencing technologies each provide distinct and complementary perspectives on the microbiological composition of carious dentin; however, none offers a complete representation of the caries-associated microbiome. Culture-based techniques primarily capture viable and cultivable microorganisms, thereby reflecting the metabolically active fraction of the biofilm. However, their limited ability to recover fastidious and obligate anaerobic taxa results in a systematic underestimation of microbial diversity [29,37,40].

In contrast, molecular approaches, including conventional PCR and 16S rRNA sequencing, substantially broaden detectable microbial diversity by identifying cultivable and non-cultivable taxa. This expanded sensitivity enables a more comprehensive taxonomic profile, particularly in deep dentinal lesions where ecological conditions favor anaerobic and low-abundance species [1,29,40,46]. However, molecular detection is inherently limited by its inability to distinguish between viable and non-viable microorganisms, which reduces its direct clinical specificity in the context of active infection [38,42,43].

Quantitative PCR and viability-based modifications (e.g., PMA-qPCR) attempt to bridge this gap by estimating bacterial load or excluding DNA from non-viable cells, but these methods remain methodologically sensitive and not fully standardized for routine clinical use [42,43]. Similarly, high-throughput sequencing and metagenomic approaches provide broader ecological and functional insights yet introduce additional interpretative complexity due to bioinformatic variability and lack of standardized analytical pipelines [41,46,47,48].

Overall, the observed differences between methodologies should not be interpreted as contradictory findings but rather as reflections of different analytical resolutions of the same biological system. Culture-based methods reflect viability, PCR-based methods reflect genetic presence, and sequencing approaches reflect community structure and functional potential. Accordingly, an integrated methodological framework combining culture-dependent and molecular techniques is necessary to achieve a clinically meaningful interpretation of the caries-associated microbiome [29,37,47,48].

## 5. Microbiological Impact of Minimally Invasive Caries Excavation Techniques

Contemporary minimally invasive dentistry is founded on the principle of preserving healthy tooth structure to the greatest extent possible while selectively removing infected dentin and retaining affected dentin with remineralization potential. Within this biologically oriented paradigm, increasing emphasis is placed on the control of residual microbial activity, effective cavity sealing, and the stabilization of the local ecological environment [50,51].

Microbiological outcomes following excavation vary considerably depending on the technique employed, lesion depth, patient age, and the microbiological assessment methods used. Culture-based and molecular studies have demonstrated that residual microorganisms may be detected following all minimally invasive approaches, even when the clinical criteria for complete excavation have been fulfilled [29,37].

### 5.1. Conventional Mechanical Excavation

Conventional mechanical excavation using rotary instruments remains the most widely employed method for removing carious dentin. Its principal advantage lies in its speed and effectiveness in reducing the bacterial load. Several culture-based studies have demonstrated a significant reduction in *Streptococcus mutans* and *Lactobacillus* spp. following mechanical excavation, particularly in shallow and moderately deep lesions [29,52].

Nevertheless, in deep carious lesions, aggressive mechanical excavation increases the risk of pulp exposure and the unnecessary removal of affected dentin with remineralization potential [51]. Bjørndal et al. demonstrated that selective carious tissue removal results in a substantial reduction in bacterial load without requiring complete sterilization of the cavity [35]. Similar findings have been reported for partial caries removal techniques, in which the residual microbiota gradually loses metabolic activity following hermetic restoration of the cavity [14].

Furthermore, molecular analyses have shown that anaerobic and difficult-to-culture taxa frequently persist after mechanical excavation, including *Prevotella*, *Veillonella*, and *Fusobacterium*, which are not consistently detected using conventional culture methods [21,37].

### 5.2. Chemo-Mechanical Excavation

Chemo-mechanical excavation techniques have been developed to selectively dissolve degraded collagen while minimizing mechanical trauma to healthy dentin. The most extensively investigated chemo-mechanical systems are Carisolv (MediTeam AB, Göteborg, Sweden), Papacárie Duo (Formula & Ação, São Paulo, Brazil), and the more recently introduced papain-based agent BRIX 3000 (Brix Medical Science, Carcarañá, Argentina). Carisolv is one of the earliest chemo-mechanical systems; it is based on sodium hypochlorite and amino acid gel chemistry and has been evaluated in multiple clinical studies for selective removal of infected dentin. Papacárie, a papain-based gel, has been investigated in pediatric and minimally invasive dentistry due to its biological selectivity and minimal discomfort during application. Clinical and microbiological studies evaluating Carisolv, Papacárie, and BRIX 3000 have demonstrated effective reduction in cariogenic microorganisms while maintaining a conservative excavation approach [53,54,55]. Similar findings have been reported in studies evaluating BRIX 3000 in primary molars, where fluorescence analysis revealed a significant reduction in bacterial load [33].

Among the most advanced products, BRIX 3000 has attracted considerable attention due to its high papain concentration and biologically conservative mechanism of action [50,51]. However, similar biological and clinical outcomes have also been reported for Carisolv and Papacárie in minimally invasive caries management, supporting the class-wide effectiveness of chemo-mechanical approaches.

Furthermore, an 18-month prospective clinical follow-up demonstrated the method’s favorable clinical stability and high biological tolerance in pediatric patients [56]. Several comparative studies evaluating mechanical and chemo-mechanical excavation have reported similar levels of bacterial reduction; however, chemo-mechanical approaches were associated with significantly greater patient comfort and lower anxiety levels in children [55,56,57,58,59,60].

### 5.3. Laser-Assisted Excavation

Laser technologies, including erbium-doped yttrium aluminum garnet (Er:YAG) and erbium, chromium-doped yttrium scandium gallium garnet (Er,Cr:YSGG) lasers, have been investigated as alternatives to conventional excavation due to their ability to selectively ablate demineralized tissues and their potential antimicrobial effects [61,62].

Several in vitro and clinical studies have demonstrated a significant reduction in viable microorganisms following laser treatment, including *Streptococcus mutans* and *Lactobacillus* spp. [63,64]. The high temperatures generated during laser irradiation, together with photoacoustic effects, may contribute to bacterial cell destruction and biofilm disruption.

Despite these promising findings, laser-assisted approaches have several limitations, including high costs, the need for specialized equipment, and variable application parameters. Furthermore, long-term clinical studies comparing microbiological outcomes with those achieved using conventional minimally invasive techniques are lacking [61,65].

### 5.4. Photodynamic Therapy

Photodynamic therapy (PDT) is an adjunctive antimicrobial approach in which a photosensitizer is activated with light of a specific wavelength, generating reactive oxygen species with bactericidal activity [66,67,68].

Recent studies have demonstrated that PDT can significantly reduce the residual microflora following selective excavation, particularly when combined with chemo-mechanical approaches [69,70,71]. Systematic reviews and controlled clinical studies have reported that adjunctive PDT significantly reduces bacterial counts in carious dentin, particularly when combined with minimally invasive excavation techniques. Nevertheless, the magnitude of the antimicrobial effect varies according to the photosensitizer, light source, and treatment protocol employed. Consistent with these observations, our study demonstrated that the combination of BRIX 3000 and photodynamic therapy resulted in a greater reduction in cariogenic bacteria compared with chemo-mechanical excavation alone, corroborating previous reports on the antimicrobial efficacy of adjunctive PDT [33,69,70,71].

PCR-based analyses have shown that PDT reduces the levels of *Streptococcus mutans* and *Lactobacillus* spp.; however, it does not always eliminate anaerobic taxa inside deep dentin [70,71]. These findings underscore that even adjunctive antimicrobial strategies do not ensure complete sterilization of the lesion.

### 5.5. Differences Between Primary and Permanent Teeth

Microbiological outcomes following minimally invasive excavation may differ between primary and permanent teeth due to variations in dentin structure, the degree of mineralization, and dentinal tubule thickness [72,73]. Primary teeth generally exhibit more rapid caries progression and greater dentin porosity, which may facilitate bacterial invasion [74,75].

Several pediatric studies have shown that chemo-mechanical approaches and adjunctive photodynamic therapy (PDT) techniques may be particularly beneficial in primary teeth because of their minimally invasive nature and better acceptance by children [32,33,56].

This supports the contemporary concept that clinical success depends primarily on the biological control of the residual microflora and the quality of the definitive restoration rather than on the achievement of absolute dentin sterility [25,54].

Accordingly, the available evidence suggests that minimally invasive excavation techniques should be evaluated based on their capacity to reduce and biologically control the residual microbial burden, not their ability to achieve complete dentin sterility.

A comparative summary of the microbiological outcomes and clinical characteristics of the principal minimally invasive excavation techniques is presented in Table 4.

Although all minimally invasive excavation techniques can significantly reduce microbial burden, their clinical relevance cannot be determined solely based on microbiological reduction. Instead, each method represents a trade-off between tissue preservation, patient-centered outcomes, procedural complexity, and residual microbial ecology. Selective caries removal and chemo-mechanical excavation provide the most balanced clinical profile, combining sufficient microbial reduction with preservation of affected dentin [26,54]. In contrast, laser-assisted and photodynamic approaches function primarily as adjunctive modalities that enhance microbial reduction but lack sufficient evidence to support their independent clinical superiority [59,68,71].

Nevertheless, current evidence suggests that residual microbial reduction combined with effective cavity sealing plays a critical role in the clinical performance of strategies for minimally invasive caries management.

## 6. Integration of Culture-Based and Molecular Findings

The assessment of microbial communities within carious dentin has revealed substantial discrepancies between culture-based and molecular diagnostic approaches, reflecting methodological limitations and the inherent ecological complexity of the caries process. Culture-based methods primarily identify viable and rapidly growing bacterial populations, most commonly members of *Streptococcus* spp. and *Lactobacillus* spp. Meanwhile, PCR-based and 16S rRNA sequencing analyses consistently reveal a considerably broader microbial diversity, including obligate anaerobes and difficult-to-culture taxa [29,37,39,40].

This discrepancy should not be interpreted solely as an analytical limitation but rather as a reflection of the so-called “method-dependent microbiome”, whereby the observed microbial profile is determined by the lesion’s actual biological composition and the diagnostic technique employed. In deep carious dentin, this effect is further amplified by the simultaneous presence of metabolically active bacteria, dormant cellular forms, and residual bacterial DNA incorporated within the demineralized collagen matrix [38,46,49].

Culture-based methods provide information regarding the viable and potentially infectious fraction of the microbiome, as they reflect only microorganisms capable of growing under laboratory conditions. In contrast, molecular techniques capture a broader ecological signature, encompassing viable and non-viable bacterial components, which may lead to an overestimation of the active infectious burden when the findings are interpreted in isolation [42,43,48,76].

An integrated analysis of both approaches indicates that carious dentin represents a dynamic and spatially heterogeneous ecosystem. The lesion’s superficial layers are more frequently associated with metabolically active and acidogenic microbial communities, whereas the deeper regions contain a mixture of anaerobic consortia, reduced metabolic activity, and structurally compromised microbial remnants [1,45,77].

From a clinical perspective, these observations help explain the persistence of microbial signals following minimally invasive excavation [77,78,79]. The persistence of bacterial DNA or low-virulence microorganisms does not necessarily indicate therapeutic failure, provided that ecological conditions are altered in a manner that suppresses metabolic activity and interrupts biofilm progression [42,48].

Therefore, discrepancies between culture-based and molecular findings should be regarded as complementary rather than conflicting. Their integration reinforces a broader ecological interpretation of caries management [1,37].

## 7. Clinical Implications for Minimally Invasive Caries Management

The integration of culture-based and molecular data in the analysis of carious dentin has direct implications for clinical decision making in contemporary minimally invasive dentistry. Rather than pursuing complete microbial elimination, the clinical focus has shifted toward disease control through the selective removal of infected tissues and the stabilization of residual microbial activity [7].

The choice of excavation technique should be individualized according to lesion depth, the tooth’s anatomical characteristics, and the risk of pulp exposure. In shallow and moderately deep lesions, conventional mechanical excavation remains an effective method for rapidly reducing the bacterial load. In deep carious lesions, however, selective or stepwise carious tissue removal is preferred, as it allows the preservation of dentin with remineralization potential and reduces the risk of iatrogenic complications [80].

Chemo-mechanical approaches, including papain-based products, provide a biologically conservative alternative for carious tissue removal. Simultaneously, they achieve a substantial reduction in cariogenic microflora. These techniques are particularly suitable for pediatric dentistry, where patient comfort and behavioral acceptance are important determinants of therapeutic success [32,33,58].

Laser-assisted approaches and photodynamic therapy may further reduce the residual microbial burden. Their benefits are most relevant in deep and difficult-to-access lesions. However, current evidence does not support their use as independent treatment approaches. Instead, they should be considered adjunctive strategies within a combined therapeutic approach [34,63,64,65].

Differences between primary and permanent teeth further emphasize the need for an individualized therapeutic approach. In primary teeth, greater dentin porosity and more rapid lesion progression require a careful balance between tissue preservation and infection control. In permanent teeth, the therapeutic focus is generally on long-term biological stability and tooth preservation [81].

In summary, the contemporary management of deep carious lesions follows a biologically oriented approach. Clinical success can be achieved using minimally invasive techniques and effective restoration, without the need for complete dentin sterility.

## 8. Future Perspectives

Contemporary research on carious dentin is gradually shifting from descriptive microbiology to a functional and ecological interpretation of the oral microbiome. Future studies should, therefore, aim for higher-resolution microbial analyses. Moreover, they should better distinguish metabolically active populations and clarify the relationship between microbiological data and clinical outcomes [7,15,46].

One promising direction is the refinement of 16S rRNA sequencing by analyzing different hypervariable regions, such as V1–V2 and V3–V4. Another important step is the adoption of full-length 16S sequencing technologies. These approaches allow more accurate taxonomic classification and reduce errors in the identification of closely related species. This is particularly relevant in carious dentin, where many anaerobic taxa show functional similarities but may have distinct pathogenic roles [26,39].

In parallel, shotgun metagenomics is emerging as the next step in oral microbiome research. Unlike targeted PCR and 16S-based approaches, it enables taxonomic and functional characterization of microbial communities. It also provides information on metabolic pathways involved in acid production, biofilm formation, proteolytic activity, and adaptation to low-pH environments. These processes play a central role in caries progression [3,45].

Another important area of development is the distinction between microbial composition and microbial activity. Metatranscriptomic analyses remain relatively uncommon in dental research. However, they can potentially identify metabolically active processes within the biofilm, which may allow for a more accurate assessment of the pathogenic potential of residual microflora after treatment [37,43].

Future research is also expected to increasingly adopt integrative multi-omics approaches that combine genomics, transcriptomics, and metabolomics. They can provide information about the composition of microbial communities and their functional dynamics. Such insights may improve our understanding of different stages of carious lesion development and the microbial response to treatment approaches [44].

From a clinical perspective, future studies will likely focus on biomarkers of caries activity. These biomarkers could improve the prediction of lesion progression and support personalized minimally invasive treatment. They may also facilitate risk stratification and help guide the selection of the most appropriate excavation strategy [1,22].

Although the findings from pilot and experimental studies are promising, the integration of these technologies into routine clinical practice remains limited. High costs, analytical complexity, and the lack of standardized protocols continue to represent major barriers. Nevertheless, the overall trend is clear: caries microbiology is moving beyond culture-based descriptions toward functional, ecological, and personalized diagnostic models [18,75].

## 9. Conclusions

In summary, this review highlights that carious dentin is a highly dynamic and heterogeneous microbial ecosystem, where disease progression is driven by ecological shifts rather than the presence of a single dominant pathogen. Culture-based and molecular approaches provide complementary but inherently biased perspectives. Molecular techniques reveal a broader microbial diversity, while culture-based methods remain essential for assessing viable and clinically relevant microorganisms.

Across minimally invasive caries excavation techniques, complete microbial elimination is rarely achieved. However, substantial microbial reduction combined with effective cavity sealing is sufficient to achieve clinical success. These findings support a biologically oriented approach to caries management, in which ecological control is prioritized over sterilization.

Advances in high-resolution sequencing technologies and integrative multi-omics approaches will provide deeper insights into the interactions between the microbiome, dentin structure, and treatment approaches. This knowledge will support the development of next-generation minimally invasive dental care.

## Figures and Tables

**Table 1:** The principal microorganisms associated with carious dentin and their associated features.

Microorganism	Key Characteristics	Putative Ecological Role in Caries Progression	Typical Association
*S. mutans*	Acidogenic; aciduric facultative anaerobe	Associated with early enamel and dentin demineralization via acidogenic activity	Early active carious lesions
*Lactobacillus* spp.	Highly acid-tolerant; fermentative	Associated with lesion progression and persistence in low-pH environments	Deep dentinal carious lesions
*Actinomyces* spp.	Facultative anaerobes; biofilm-forming taxa	Associated with chronic infection and root caries development	Root and dentinal carious lesions
*Prevotella* spp.	Anaerobic; proteolytic taxa	Associated with organic matrix degradation in advanced lesions	Deep carious dentin
*Veillonella* spp.	Lactate-utilizing anaerobes	Involved in metabolic cross-feeding within biofilms	Mixed-species biofilms
*Fusobacterium* spp.	Obligate anaerobes; bridging organisms	Associated with biofilm co-aggregation and community structuring	Advanced lesions
*Scardovia wiggsiae*	Acidogenic anaerobic bacterium	Frequently associated with severe caries, particularly early-childhood caries	Severe/ECC lesions
*Bifidobacterium* spp.	Acidogenic anaerobes detected in caries lesions	Reported in association with acidified ecological niches	Progressing lesions
*Candida albicans*	Opportunistic fungal species	Reported in association with bacterial biofilms and synergistic interactions	Severe caries cases (reported)

**Table 2:** Culture-based studies evaluating microbiological outcomes of caries excavation techniques in dentinal lesions.

Study	Design/Material	Excavation Method	Microbiological Findings	Clinical Relevance
Lager et al., 2003 [11]	Deep dentinal lesions	Conventional bur vs. Carisolv	Residual cultivable bacteria detected in both groups	Complete sterility rarely achieved
Toi et al., 2003 [31]	Extracted permanent molars	Hand excavation (ART)	Persistent bacteria in dentinal tubules after excavation	Clinical hardness does not guarantee sterility
Bjørndal et al., 1998 [35]	Stepwise excavation	Selective excavation	Significant bacterial reduction after sealing	Supports minimally invasive concept
Singhal et al., 2016 [14]	Primary teeth RCT	Partial vs. complete caries removal	Similar microbial reduction among methods	Partial excavation clinically acceptable
Ferreira et al., 2023 [12]	Systematic review	Antiseptics + chemo-mechanical methods	Heterogeneous antimicrobial outcomes	Lack of consensus persists
Lazarova et al., 2025 [32]	Primary teeth	BRIX 3000	Significant reduction in cariogenic bacteria	Effective minimally invasive approach
Lazarova et al., 2026 [33]	Primary teeth	BRIX + PDT	Additional antimicrobial effect	Adjunctive PDT improves reduction
Mitova et al., 2026 [34]	Permanent teeth	Culture vs. PCR	PCR detected broader microbial spectrum	Culture underestimates diversity

**Table 3:** Comparative characteristics of culture-based and molecular methods for analysis of carious dentin microbiota.

Method	Detects	Advantages	Limitations	Findings	Clinical Relevance
Culture-based methods	Viable and cultivable bacteria	Viability + susceptibility testing	Underestimates diversity; anaerobe bias	*Streptococcus* and *Lactobacillus* dominance	Viable load after excavation
Conventional PCR	Specific bacterial DNA sequences	High sensitivity; rapid	No viability distinction	Cariogenic taxa detection (*Prevotella*, *Veillonella*, and *Fusobacterium*)	Targeted pathogen identification
Quantitative PCR (qPCR)	Quantitative DNA	Semi-quantification	Limited targets; DNA persistence	Residual bacterial load	Monitoring reduction
16S rRNA sequencing	Taxonomic profile	Broad coverage; uncultivable taxa	Limited function; bioinformatics load	Diverse anaerobic consortia	Ecological profiling
Shotgun metagenomics	Taxonomy + genes	Functional pathways; high resolution	Cost; complexity	Virulence; acid/biofilm genes	Advanced research use
Viability PCR (PMA-qPCR)	Viable-cell DNA	Live/dead differentiation	Standardization/variability issues	Residual viable microbiota	Viability-focused assessment
Metatranscriptomics	Microbial gene expression	Active metabolism profiling	RNA instability	Active biofilm pathways	Experimental functional insight

**Table 4:** Microbiological outcomes associated with minimally invasive excavation techniques in carious dentin.

Method	Microbiological Effect	Advantages	Limitations
Conventional mechanical excavation	Substantial reduction in cultivable bacteria	Fast, effective, and widely available	Risk of over-excavation and pulp exposure
Selective/partial caries removal	Moderate reduction in microbial load with residual microbiota often present	Preserves affected dentin; reduced risk of pulp exposure	Viable bacteria may persist in deep dentin
Chemo-mechanical excavation	Significant reduction in cariogenic bacteria	Minimally invasive; improved patient comfort	Longer clinical time
Laser-assisted excavation	High reduction in viable microorganisms (variable depending on parameters)	Selective tissue removal; potential biofilm disruption	High cost; lack of standardization
Photodynamic therapy (PDT) (adjunctive)	Additional reduction in residual microbiota when used with other methods	Enhances antimicrobial effect	Limited efficacy as standalone treatment
Combined chemo-mechanical excavation + PDT	Greater microbial reduction compared with chemo-mechanical treatment alone	Potential synergistic antimicrobial effect	Limited long-term clinical evidence

## Data Availability

No new data were created or analyzed in this study.

## Abbreviations

The following abbreviations are used in this manuscript:

PCR	Polymerase Chain Reaction
qPCR	Quantitative Polymerase Chain Reaction
PMA-qPCR	Propidium Monoazide–Quantitative Polymerase Chain Reaction
DNA	Deoxyribonucleic Acid
16S rRNA	16S Ribosomal Ribonucleic Acid
Er:YAG	Erbium-Doped Yttrium Aluminum Garnet (Laser)
Er,Cr:YSGG	Erbium, Chromium-Doped Yttrium Scandium Gallium Garnet (Laser)
ART	Atraumatic restorative treatment
PDT	Photodynamic therapy
